# Increased caveolin-1 in intervertebral disc degeneration facilitates repair

**DOI:** 10.1186/s13075-016-0960-y

**Published:** 2016-03-03

**Authors:** Frances C. Bach, Ying Zhang, Alberto Miranda-Bedate, Lucy C. Verdonschot, Niklas Bergknut, Laura B. Creemers, Keita Ito, Daisuke Sakai, Danny Chan, Björn P. Meij, Marianna A. Tryfonidou

**Affiliations:** Department of Clinical Sciences of Companion Animals, Faculty of Veterinary Medicine, Utrecht University, Utrecht, The Netherlands; Department of Biochemistry, The University of Hong Kong, Hong Kong, China; Department of Orthopaedics, University Medical Center Utrecht, Utrecht, The Netherlands; Orthopaedic Biomechanics, Department of Biomedical Engineering, Eindhoven University of Technology, Eindhoven, The Netherlands; Department of Orthopaedic Surgery, Tokai University School of Medicine, Isehara, Japan

**Keywords:** Caveolin-1, Intervertebral disc, Degeneration, Nucleus pulposus, Back pain, Canine, Human, Mice

## Abstract

**Background:**

Preceding intervertebral disc (IVD) degeneration, the cell phenotype in the nucleus pulposus (NP) shifts from notochordal cells (NCs) to chondrocyte-like cells (CLCs). Microarray analysis showed a correlation between caveolin-1 expression and the phenotypic transition of NCs to CLCs. With a clinical directive in mind, the aim of this study was to determine the role of caveolin-1 in IVD degeneration. As a scaffolding protein, caveolin-1 influences several signaling pathways, and transforming growth factor (TGF)-β receptors have been demonstrated to colocalize with caveolin-1. Therefore, the hypothesis of this study was that caveolin-1 facilitates repair by enhancing TGF-β signaling in the IVD.

**Methods:**

Protein expression (caveolin-1, apoptosis, progenitor cell markers, extracellular matrix, and phosphorylated Smad2 [pSmad2]) was determined in IVDs of wild-type (WT) and caveolin-1-null mice and canine IVDs of different degeneration grades (immunofluorescence, immunohistochemistry, and TUNEL assay). Canine/human CLC microaggregates were treated with chondrogenic medium alone or in combination with caveolin-1 scaffolding domain (CSD) peptide and/or caveolin-1 silencing RNA. After 28 days, gene and protein expression profiles were determined.

**Results:**

The NP of WT mice was rich in viable NCs, whereas the NP of caveolin-1-null mice contained more collagen-rich extracellular matrix and fewer cells, together with increased progenitor cell marker expression, pSmad2 TGF-β signaling, and high apoptotic activity. During canine IVD degeneration, caveolin-1 expression and apoptotic activity increased. In vitro caveolin-1 silencing decreased the CLC microaggregate glycosaminoglycan (GAG) content, which could be rescued by CSD treatment. Furthermore, CSD increased TGF-β/pSmad2 signaling at gene and protein expression levels and enhanced the anabolic effects of TGF-β_1_, reflected in increased extracellular matrix deposition by the CLCs.

**Conclusions:**

Caveolin-1 plays a role in preservation of the NC phenotype. Additionally, it may be related to CLC apoptosis, given its increased expression in degenerated IVDs. Nevertheless, CSD enhanced CLC GAG deposition in vitro, and hence the increased caveolin-1 expression during IVD degeneration may also facilitate an ultimate attempt at repair. Further studies are needed to investigate how caveolin-1 modifies other signaling pathways and facilitates IVD repair.

**Electronic supplementary material:**

The online version of this article (doi:10.1186/s13075-016-0960-y) contains supplementary material, which is available to authorized users.

## Background

Low back pain has been identified as one of the highest burden conditions by the World Health Organization and is the most common type of pain restricting daily activity [1]. Low back pain is strongly related to intervertebral disc (IVD) degeneration [2]. The IVD consists of a central nucleus pulposus (NP) and outer annulus fibrosus. During IVD degeneration, the glycosaminoglycan (GAG) and water content of the NP decreases. Like humans, dogs experience spontaneous IVD degeneration with similar characteristics [3]. In both species, notochordal cells (NCs) are replaced by chondrocyte-like cells (CLCs) in the NP during maturation and early IVD degeneration. Therefore, the dog is considered to be a suitable animal model for human IVD degeneration [3]. Dogs can be divided into chondrodystrophic (CD) and nonchondrodystrophic (NCD) breeds on the basis of their physical appearance. In CD dogs, replacement of NCs by CLCs starts around 1 year of age and IVD disease typically develops around 3–7 years of age, whereas in NCD dogs, NCs remain the predominant cell type until middle or old age and if IVD disease develops it occurs around 6–8 years of age [4]. Current treatments for IVD disease (physiotherapy, medication, and surgery) are focused on reducing pain and spinal cord and/or nerve decompression but do not induce IVD repair. Therefore, there is substantial interest in agents stimulating biological repair of the degenerated IVD, resulting in functional restoration [5]. Caveolin-1 could be such an agent, as its advantageous effects have been demonstrated in several tissue types [6–9].

The mammalian caveolin family consists of three proteins (caveolin-1, -2, and -3), which are integral membrane proteins essential for the structural integrity and function of caveolae (flask-shaped invaginations of the plasma membrane) of many differentiated cell types [10–12]. Caveolin-1 is involved in endocytosis, the formation and transport of caveolae, and cell adhesion and migration [13]. Moreover, caveolin-1 is implicated in cell cycle regulation, senescence (cell cycle arrest due to resistance to external stimuli [10]), and apoptosis [14]. As a scaffolding protein, it regulates signal transduction [12], and caveolin-binding motifs have been identified in several target proteins [7]. The effect of caveolin-1 on signaling pathways is usually inhibitory due to sequestration and inactivation of molecules in caveolar membranes, but signaling can also be enhanced by bringing molecules together [7]. Altogether, caveolin-1 has been shown to exert context-dependent effects (i.e., differing per tissue/cell type, age, and stage of degeneration) [11].

Caveolin-1 is differentially expressed in the IVD. The NP of early degenerated canine IVDs expresses lower caveolin-1 levels than healthy canine NC-rich NPs [15]. In addition, the NP of wild-type (WT) mice is rich in viable NCs, whereas the NP of caveolin-1-null mice contains mainly CLCs [15]. In humans, CLCs derived from degenerated IVDs express higher caveolin-1 levels than CLCs from nondegenerated IVDs [16]. During IVD degeneration, accelerated cell senescence takes place [16, 17], and a positive relationship between caveolin-1 expression and premature stress-induced senescence has been reported in articular cartilage [18] and the IVD [16]. Altogether, this implies that caveolin-1 could play an important role in IVD degeneration.

The aim of this study was to determine the role of caveolin-1 in IVD degeneration. Transforming growth factor (TGF)-β signaling is known to play a role in the degenerative and regenerative processes of cartilaginous tissues [19], and TGF-β receptors have been demonstrated to colocalize with caveolin-1 [20]. Therefore, we hypothesized that caveolin-1 modifies canonical TGF-β signaling in the IVD and thereby facilitates repair. To assess the effect of in vivo caveolin-1 depletion, we studied murine caveolin-1-null IVDs. With a clinical directive in mind, functional studies were focused on the effect of caveolin-1 on CLCs because degenerated canine and human IVDs contain almost 100 % CLCs. Caveolin-1 expression and apoptosis levels were determined in canine IVDs of different degeneration grades, and human and canine CLCs were silenced for caveolin-1 and/or supplemented with caveolin-1 scaffolding domain (CSD) peptide in vitro to determine the (regenerative) effect of caveolin-1 on CLCs. In the present study, we show that while caveolin-1 preserved the NC phenotype, its expression increased in degenerated IVDs. Given that CSD supplementation enhanced the anabolic effects of TGF-β_1_ on CLCs in terms of increased GAG deposition in vitro, the increased caveolin-1 expression during IVD degeneration may facilitate an ultimate attempt at repair by decreasing the loss of GAGs.

## Methods

### Caveolin-1-null mice

Thoracic and lumbar spines were collected from 1.5-, 3-, and 6-month-old caveolin-1-null (*Cav*^*tm1Mls*^, JAX®; The Jackson Laboratory, Bar Harbor, ME, USA) and WT mice (B6129SF2, JAX®) of the same genetic background. Experimental procedures were performed according to the guidelines of the Utrecht University Ethics Committee (DEC 2008.III.01.001).

### Paraffin-embedded tissue histology

Murine lumbar spines were fixed in 4 % neutral buffered formaldehyde (Klinipath, Duiven, the Netherlands), decalcified (7 days in 10 % ethylenediaminetetraacetic acid), and embedded in paraffin. Midsagittal 5-μm sections were mounted on KP+ microscope slides (KP-3056; Klinipath), stained with hematoxylin and eosin and Alcian Blue/picrosirius red [21], and immunohistochemically stained for Ki-67 and collagen type II (Table 1). An ApopTag® Plus Peroxidase In Situ Apoptosis Detection Kit (terminal deoxynucleotidyl transferase deoxyuridine triphosphtate nick-end labeling [TUNEL] assay, S7101; EMD Millipore, Billerica, MA, USA) was used to detect apoptotic cells on lumbar midsagittal sections. All lumbar IVDs were evaluated (approximately four IVDs per lumbar spine). Raw images of the sections were made using a Leica DFC420C digital camera (Leica Microsystems, Buffalo Grove, IL, USA) mounted to a BX60 microscope (Olympus America, Center Valley, PA, USA) with the Leica Application Suite (V4.2) software package. Adobe Photoshop CS6 software (Adobe, San Jose, CA, USA) was used to manually count (positively stained) cell numbers in each NP. The percentage of nuclei that stained positive for TUNEL over the total number of nuclei present was determined for every murine NP in conjunction with morphology to avoid false-positive results.

**Table 1:** Details of the immunohistochemistry protocol

Target protein	Clone/catalog number (manufacturer)	Origin	Antigen retrieval	Block	Concentration of first Ab	Second Ab (catalog number; manufacturer)
Caveolin-1	Clone 2297, 610406 (BD Biosciences, San Jose, CA, USA)	Mouse	Citrate (10 mM), 70 °C, 60 minutes	0.3 % H_2_O_2_ + 10 % goat serum	5 μg/ml in PBS	Goat anti-mouse, (K4001; Dako, Carpinteria, CA, USA)
Ki-67	Clone SP6, RM-9106-S (Thermo Scientific™, Waltham, MA, USA)	Rabbit	Citrate (10 mM), 80 °C, 90 minutes	0.3 % H_2_O_2_ + 10 % goat serum	4 μg/ml in PBS	Goat anti-rabbit, (K4003; Dako)
Collagen type II	II-II6B3 (Developmental Studies Hybridoma Bank, Iowa City, IA, USA)	Mouse	1 mg/ml pronase + 10 mg/ml hyaluronidase, 37 °C, 30 minutes	0.3 % H_2_O_2_ + 5 % BSA/PBS	0.02 μg/ml (murine, canine) or 0.4 μg/ml (human) in 5 % BSA/PBS	Goat anti-mouse, (K4001; Dako)
Collagen type I	ab6308 (Abcam, Cambridge, UK)	Mouse	1 mg/ml pronase + 10 mg/ml hyaluronidase, 37 °C, 30 minutes	0.3 % H_2_O_2_ + 5 % BSA/PBS	0.1 μg/ml (human) or 0.07 μg/ml (canine) in 5 % BSA/PBS	Goat anti-mouse, (K4001; Dako)

*Ab* antibody, *BSA* bovine serum albumin, *PBS* phosphate-buffered saline

Midsagittal intervertebral disc sections were deparaffinized with xylene and graded ethanol. The primary antibody was applied at 4 °C overnight. In control experiments, the primary antibody was replaced with mouse immunoglobulin G1 (3877; Santa Cruz Biotechnology), and no false-positive staining was observed. The secondary antibody was applied for 60 minutes, and the sections were incubated with a liquid 3,3′-diaminobenzidinesubstrate chromogen system (K3468; Dako) for 10 minutes. The slides were stained with Vector Hematoxylin QS solution (H3404; Vector Laboratories, Burlingame, CA, USA) for 10 seconds, dehydrated, and mounted (H5000; Vector Laboratories)

### Immunofluorescent staining

Thoracic IVDs were snap-frozen and stored at −70 °C until analysis. Ten-micrometer transverse cryosections were stained for caveolin-1, phosphorylated Smad2 (pSmad2 indicating activated TGF-β signaling), and the nucleus pulposus progenitor cell (NPPC) markers tyrosine-like receptor Tie2 and disialoganglioside 2 (GD2) (Table 2). Within 48 h, confocal images were obtained with a Zeiss LSM upright confocal laser scanning microscope, and the sections were analyzed with Zen software 2011 (Zeiss Microscopy, Thornwood, NY, USA) in two or three transverse thoracic NP sections per animal. On the basis of the observed expression pattern, caveolin-1, Tie2, and GD2 were scored as being present or not present. The percentage of nuclei that stained positive for pSmad2 over the total number of nuclei present (pSmad2 ratio) was manually counted and calculated per murine NP, and the mean pSmad2 ratio was determined per age group.

**Table 2:** Details of the immunofluorescence protocol

Antibody	Specifications	Host	Dilution	Secondary antibody
Tie2	324 (Santa Cruz Biotechnology)	Rabbit	4 μg/ml	Alexa Fluor® 594 AffiniPure Donkey Anti-Rabbit IgG (711-165-152; Jackson ImmunoResearch, West Grove, PA, USA)
GD2	554272 (BD Biosciences)	Mouse	10 μg/ml	Alexa Fluor™ 488 Donkey Anti-Mouse IgG (A21202; Molecular Probes/Invitrogen, Eugene, OR, USA)
Caveolin-1	2910 (Abcam)	Rabbit	5 μg/ml	Alexa Fluor® 594 AffiniPure Donkey Anti-Rabbit IgG (711-165-152; Jackson ImmunoResearch)
pSMAD2	3101 s (Cell Signaling Technology, Danvers, MA, USA)	Rabbit	5 μg/ml	Alexa Fluor™ 488 Donkey Anti-Rabbit IgG (A21206; Molecular Probes/Invitrogen)
Negative control	Primary antibody omitted	–	–	Alexa Fluor™ 488 Donkey Anti-Mouse IgG (A21202; Molecular Probes/Invitrogen) + Alexa Fluor® 594 AffiniPure Donkey Anti-Rabbit IgG (711-165-152; Jackson ImmunoResearch)

*GD2* disialoganglioside 2, *IgG* immunoglobulin G, *pSmad2* phosphorylated Smad2

The thoracic intervertebral discs were embedded in O.C.T. compound (14020108926; Leica Microsystems), transverse 10-μm cryosections were mounted on Superfrost Plus slides (4951PLUS4; Thermo Scientific), and the sections were treated with phosphate-buffered saline (PBS) diluted 1:1 in Gibco TrypLE™ Express Enzyme (12605; Life Technologies, Carlsbad, CA, USA) for 10 minutes at 37 °C. The sections were blocked with 3 % bovine serum albumin (BSA) and 0.25 % Triton X-100 (T8787; Sigma-Aldrich, St. Louis, MO, USA) for 30 minutes and incubated overnight at 4 °C with the primary antibodies diluted in 1 % BSA in PBS. The secondary antibody was diluted 1:250 in 1 % BSA and 0.1 % Triton X-100 in PBS and applied for 45 minutes at room temperature. The sections were mounted with VECTASHIELD mounting medium with 4′,6-diamidino-2-phenylindole (H-1200; Vector Laboratories)

### Caveolin-1 expression in canine IVD degeneration

Thirty-seven thoracolumbar or lumbosacral IVD samples from sixteen canine cadavers were studied. The dogs were of different breeds (5 CD, 11 NCD), ages (1–16 years), and sex (11 female, 5 male). The samples were divided into five different grades of degeneration on the basis of gross morphology of midsagittal sections, ranging from Thompson grade I for healthy to Thompson grade V for end-stage degeneration [22, 23]. The IVD donors were chosen on the basis of equal representation of all IVD degeneration grades (*n* = 8, 7, 8, 7, and 7 for grades I–V, respectively). All dogs had been killed in unrelated research projects or were client-owned dogs that were submitted to the Department of Pathobiology Faculty of Veterinary Medicine, Utrecht University, for necropsy. Five-micrometer-thick midsagittal consecutive IVD sections were mounted on KP+ microscope slides and processed for caveolin-1 immunohistochemistry (Table 1) and a TUNEL assay. Thereafter, raw images of the sections were made using a Leica DFC420C digital camera mounted to a BX60 microscope and the Leica Application Suite (V4.2) software package. Adobe Photoshop CS6 was used to manually count (positively stained) TUNEL cell numbers, and the integrated option “color range” was used to determine the area within the NP that stained positive for caveolin-1 (caveolin-1 ratio) in four randomly selected NP areas per IVD section. The mean percentage of nuclei that stained positive for TUNEL over the total number of nuclei present in the four randomly selected NP areas was determined in conjunction with morphology to avoid false-positive results.

### In vitro effects of caveolin-1 peptide on human and canine CLCs derived from degenerated IVDs

#### CLC collection

In total, IVD tissue from 16 canine (11 CD, 5 NCD) and 3 human donors was collected. Complete spines (2–11 years of age, Thompson grade II or III) were collected from dogs that had been killed in unrelated research studies (approved by the Utrecht University Animal Ethics Committee). IVD tissue of three human donors (one male, two females, 47–63 years of age, Thompson grade III) derived from the L2–L5 spinal region were obtained at autopsy. Anonymous use of redundant tissue for research purposes is a standard treatment agreement with patients in University Medical Centre Utrecht (local medical Ethics committee number 12-364). Thus, all necessary consent from patients involved in the study was present. The material was collected as part of the activities of the tissue bank of the Department of Pathology, UMCU Biobank, UMC Utrecht. The scientific committee of the Department of Pathology at UMC Utrecht (Wetenschappelijke Adviesraad Biobank) approved the study. All tissue was used in line with the code “Proper Secondary Use of Human Tissue” as installed by the Dutch Federation of Biomedical Scientific Societies [24].

#### Cell culture and experimental design

CLCs were collected from the canine and human NPs as described previously [25] and thereafter expanded in expansion medium [26]. NCD canine donors that contained only CLCs in the NP were selected using cytospins (Additional file 1). After the CLCs reached 80 % confluence in P2, they were plated in a 96-well plate (CLS7007, Costar®; Corning, Corning Life Sciences, NY, USA) at a density of 35,000 cells/well in 50 μl of chondrogenic culture medium composed of high-glucose Dulbecco’s modified Eagle’s medium + GlutaMAX (31966; Invitrogen, Carlsbad, CA, USA) with 1 % P/S (P11-010; GE Healthcare Life Sciences, Little Chalfont, UK), 1 % ITS+ premix (354352; Corning Life Sciences), 0.04 mg/ml L-proline (P5607; Sigma-Aldrich, St. Louis, MO, USA), 0.1 mM ascorbic acid 2-phosphate (A8960; Sigma-Aldrich), 1.25 mg/ml human serum albumin (human CLCs; Sanquin, Amsterdam, the Netherlands) or bovine serum albumin (canine CLCs, A9418; Sigma-Aldrich), and 10 ng/ml TGF-β_1_ (240-B; R&D Systems, Minneapolis, MN, USA). The microaggregates were cultured for 14 days (21 % O_2_, 5 % CO_2_, 37 °C).

Caveolin-1 expression was silenced in CLCs from six CD canine donors, and the following conditions were tested: mock (100 nM Stealth™ RNAi Negative Control Duplex; Invitrogen), silencing RNA (siRNA) (100 nM caveolin-1 Stealth™ RNAi siRNA Duplex; Invitrogen), and siRNA + CSD (100 nM caveolin-1 siRNA oligo and 10 μM CSD, ALX-153-064; Enzo Life Sciences, Farmingdale, NY, USA). The canine CLCs were transfected with 3 μl/ml Lipofectamine™ RNAiMAX (13778-075; Invitrogen) at the moment of plating. Six hours after plating, the 96-well plates were centrifuged at 50 *g* for 5 minutes to induce microaggregate formation. Stealth™ RNAi siRNA Duplex (10620312; Invitrogen) was used to silence caveolin-1 (NM_001003296): 5′ CACACCAAGGAAAUCGACCUGGUCA 3′ (guanine-cytosine [GC] percentage 52 %). Stealth™ RNAi Negative Control Duplex (GC percentage 48 %, 12935-300; Invitrogen) was used to determine the effect of Stealth™ RNAi siRNA Duplex versus background (mock).

To determine the optimal conditions for CSD to exert its effects, CLCs from three human donors as well as five CD and five NCD canine donors with the following conditions were studied: T_2_ (chondrogenic culture medium with 2 ng/ml TGF-β_1_), T_2_C_10_ (2 ng/ml TGF-β_1_ and 10 μM CSD), T_2_C_25_ (2 ng/ml TGF-β_1_ and 25 μM CSD), T_10_ (10 ng/ml TGF-β_1_), T_10_C_10_ (10 ng/ml TGF-β_1_ and 10 μM CSD), and T_10_C_25_ (10 ng/ml TGF-β_1_ and 25 μM CSD).

#### Readout parameters

Two microaggregates per donor and condition were snap-frozen at day 4, and RNA was extracted, cDNA was made, and real-time quantitative polymerase chain reaction was performed for the target genes aggrecan (*ACAN*), a disintegrin and metalloproteinase with thrombospondin motifs 5 (*ADAMTS5*), activin receptor-like kinase 1 (*ALK1*), activin receptor-like kinase 5 (*ALK5*), BCL2-associated protein (*BAX*), B-cell CLL/lymphoma 2 (*BCL2*), caspase 3 (*CASP3*), caveolin-1 (*CAV1*), cyclin D1 (*CCND1*), collagen type I, alpha 1 (*COL1A1*), collagen type II, alpha 1 (*COL2A1*), inhibitor of DNA binding 1 (*ID1*), matrix metalloproteinase 13 (*MMP13*), plasminogen activator inhibitor 1 (*PAI1*, a readout for activated Smad2/3 signaling [27–29]), (sex determining region Y)-box 9 (*SOX9*), and tissue inhibitor of metalloproteinase 1 (*TIMP1*), as well as the reference genes glyceraldehyde 3-phosphate dehydrogenase (*GAPDH)*, ribosomal protein S19 (*RPS19)*, hypoxanthine-guanine phosphoribosyltransferase (*HPRT*), and succinate dehydrogenase subunit A (*SDHA*) as described previously [26]. An overview of the primer pairs is given in Additional file 2. In addition, two microaggregates per condition per donor were collected at days 7 and 14, and the GAG and DNA content of the CLC microaggregates and GAG release into the culture medium were determined as described previously [26].

To determine caveolin-1 protein silencing efficiency, two snap-frozen microaggregates per condition per donor were pooled, crushed, and dissolved in radioimmunoprecipitation assay (RIPA) buffer at days 7 and 14. Caveolin-1 protein was measured using an enzyme-linked immunosorbent assay (SEA214Ca, Cloud-Clone Corp., Houston, TX, USA). Histopathological evaluation of the microaggregates was performed with Safranin O/Fast Green staining [26] and collagen types I and II immunohistochemistry (Table 1). Western blot analysis was performed as described previously for pSmad2 (Ser465/467, 60 kDa, 3101; Cell Signaling Technology, Danvers, MA, USA) and β-actin (42 kDa, pan Ab-5; Neomarkers, Fremont, CA, USA) [30]. CD canine CLCs (*n* = 200,000; mixture of the previously used CD canine donors) were plated per well (six-well plate, 657160, CELLSTAR®; Greiner Bio-One, Monroe, NC, USA) in expansion medium. After 5 days, the cells were treated with 3 ml of chondrogenic culture medium with or without CSD. The following conditions were tested: T_2_, T_2_C_10_, T_2_C_25_, T_10_, T_10_C_10_, and T_10_C_25_. After 24 h of treatment, protein samples were obtained and homogenized in RIPA buffer containing 0.6 mM phenylmethylsulfonyl fluoride, 17 μg/ml aprotinin, and 1 mM sodium orthovanadate (Sigma-Aldrich). Protein concentrations were measured using the Qubit® Protein Assay Kit (Q32851; Invitrogen) according to the manufacturer’s instructions. The mean volume of the protein bands on the blots was determined by volumetric (INT*mm^2^) band analysis using Quantity One software (Bio-Rad Laboratories, Hercules, CA, USA). The mean volume of pSmad2 was divided by the mean volume of β-actin (pSmad2/β-actin ratio) to correct for different protein concentrations applied to the membranes.

### Statistical analysis

Statistical analysis was performed using IBM SPSS version 22 (IBM Armonk, NY, USA) and RStudio (version 0.96; RStudio, Boston, MA, USA) software. All data were examined for normal distribution (Shapiro-Wilk test). Kruskal-Wallis and Mann-Whitney *U* tests were performed on nonnormally distributed data, whereas generalized linear regression models based on analysis of variance were used for normally distributed data. A Cox proportional hazards model was used for genes with undetectable expression (cycle threshold value >40). To find correlations between the caveolin-1/TUNEL ratio and IVD degeneration score, partial correlations (corrected for donor) were determined. All of the above-mentioned tests were followed by a Benjamini-Hochberg false discovery rate post hoc correction for multiple comparisons. A *p* value less than 0.05 was considered significant.

## Results

### Effect of in vivo caveolin-1 inactivation on the murine nucleus pulposus

**Fig. 1:**
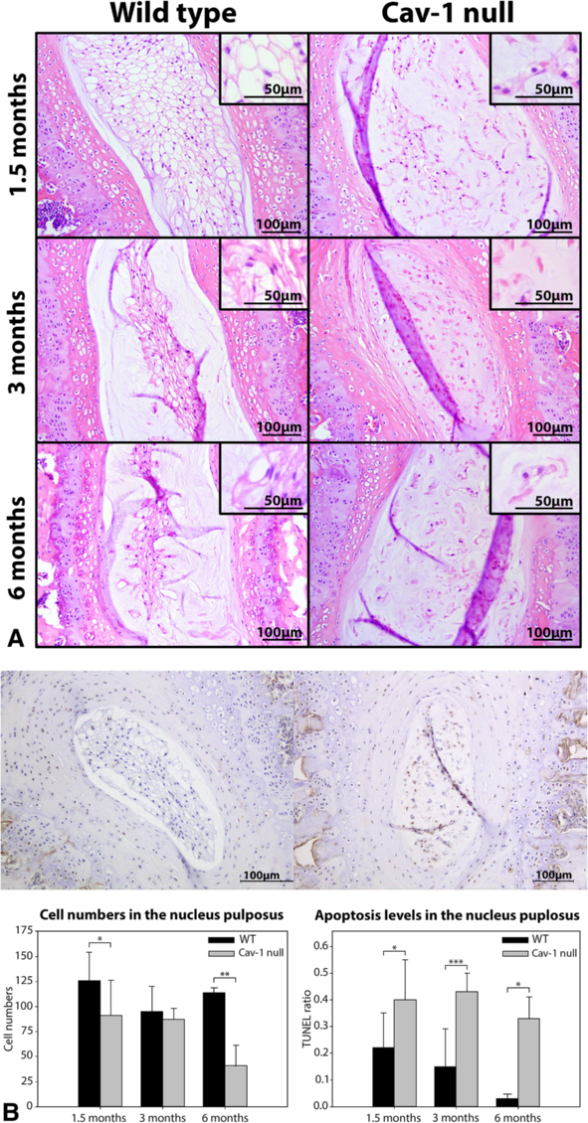
Effect of in vivo caveolin-1 inactivation on morphology and apoptosis of murine nucleus pulposus cells. **a** Hematoxylin and eosin–stained nucleus pulposus (NP) sections of wild-type (WT) and caveolin-1 (Cav-1)-null mice at 1.5, 3, and 6 months of age. Typical large, vacuolated notochordal cells (NCs) are present in the NP of WT mice, whereas smaller, nonvacuolated chondrocyte-like cells (CLCs) are present in the NP of caveolin-1-null mice. **b** The intervertebral disc (IVD) of a 1.5-month-old WT mouse stained with a terminal deoxynucleotidyl transferase deoxyuridine triphosphate nick-end labeling (TUNEL) assay shows only some brown apoptotic cell nuclei, whereas the IVD of a 1.5-month-old caveolin-1-null mouse shows many positive nuclei. NP cell numbers are significantly lower in caveolin-1-null mice than in WT mice at 1.5 and 6 months of age. The proportion of apoptotic cell nuclei (TUNEL ratio) is significantly higher in the NP of caveolin-1-null mice than in the NP of WT mice at every tested age. *p < 0.05, **p < 0.01, ***p < 0.001. *n* = 4–9 per age group and condition (WT or caveolin-1-null mice)

**Fig. 2:**
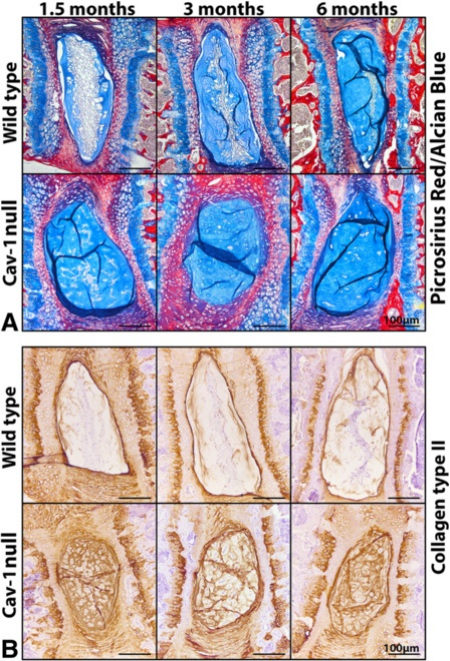
Effect of in vivo caveolin-1 (Cav-1) inactivation on extracellular matrix production of the murine nucleus pulposus (NP). Picrosirius red/Alcian Blue staining (**a**) and immunohistochemistry for collagen type II (**b**) on the NP of wild-type (WT) and Cav-1-null mice. GAGs and collagen type II are abundantly present in the extracellular matrix of Cav-1-null mice NPs, whereas they were present to a lesser extent in WT mice NPs. Bars indicate 100 μm. *n* = 4–9 per age group and condition (WT or Cav-1-null mice)

In vivo caveolin-1 loss did not affect the morphometry of the murine IVDs (Additional file 3). However, there was a distinct difference in morphology between NP cells of WT and caveolin-1-null mice. The NP of WT mice was rich in large, vacuolated NCs, whereas the NP of caveolin-1-null mice contained relatively few, smaller, nonvacuolated chondrocyte- and fibroblast-like cells at every studied age (Fig. 1a). NCs were diffusely scattered throughout the NP of 1.5-month-old WT mice, whereas they were located mainly in the center of the NP, surrounded by a rim of chondroid matrix in 3- and 6-month-old WT mice. The NP cell numbers in WT mice did not change considerably during aging, whereas NP cell numbers of caveolin-1-null mice decreased over time with significantly fewer cells at 1.5 and 6 months of age (*p* < 0.05) (Fig. 1b). At the ages tested, the proportion of positively stained TUNEL cell nuclei (TUNEL ratio) was significantly higher in the NP of caveolin-1-null mice than in WT mice (*p* < 0.05) (Fig. 1b), indicating that the in vivo loss of caveolin-1 induced apoptosis of murine NP cells, leading to lower cell numbers. In vivo caveolin-1 loss did not affect the expression of the cell proliferation–associated protein Ki-67; there was no positive Ki-67 staining in WT or caveolin-1-null mice NPs (data not shown). In the caveolin-1-null mice, GAGs and collagen type II were abundantly present in the extracellular matrix (ECM), whereas they were present to a lesser extent in WT NPs (Fig. 2).

**Fig. 3:**
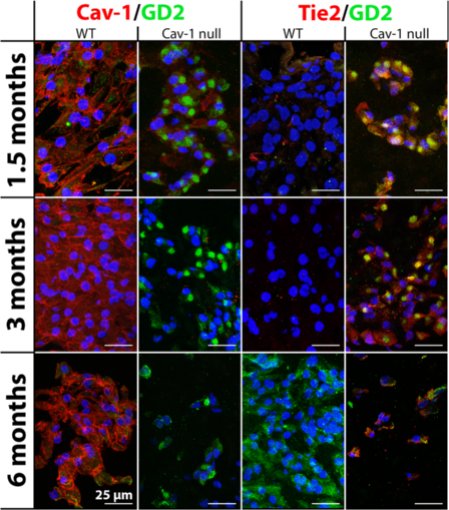
Disialoganglioside 2 (GD2) and Tie2 expression is increased in caveolin-1 (Cav-1)-null mice. Immunofluorescent staining for Cav-1 (*red*), GD2 (*green*), and Tie2 (*red*) on the nucleus pulposus of wild-type (WT) and Cav-1-null mice. The cell nuclei were stained with 4′,6-diamidino-2-phenylindole (*blue*). Bars indicate 25 μm. *n* = 2–5 per age group and condition (WT or Cav-1-null mice)

**Fig. 4:**
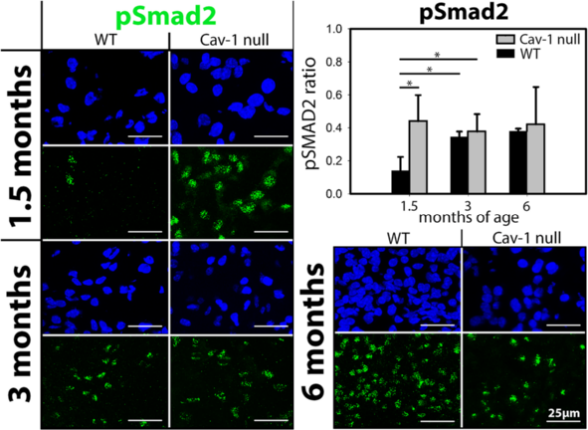
Phosphorylated Smad2 (pSmad2) expression is lower in young wild-type (WT) than in young caveolin (Cav)-1-null mice. Immunofluorescent staining for pSmad2 (*green*) on the nucleus pulposus (NP) of WT and Cav-1-null mice. The cell nuclei were stained with 4′,6-diamidino-2-phenylindole (*blue*). pSmad2 protein expression (pSmad2 ratio) is significantly lower in the murine WT NP at 1.5 months of age than in the murine WT NP at 3 months of age and the murine Cav-1-null NP at 1.5 and 3 months of age. Bars indicate 25 μm. *n* = 2–5 per age group and condition (WT or Cav-1-null mice). *p < 0.05

Immunofluorescent staining confirmed that caveolin-1-null NP cells expressed no caveolin-1 at the protein level, whereas NCs of WT mice abundantly expressed caveolin-1 in their cytoplasm and cell membranes at every studied age (Fig. 3). In vivo caveolin-1 inactivation increased the expression of NPPC markers Tie2 (differentiation marker for dormant NPPCs [31]) and GD2 (NPPC proliferation marker [31]). Caveolin-1-null NP cells abundantly expressed these markers at every studied age, whereas WT NCs expressed Tie2 only at 1.5 months of age and GD2 at 1.5 and 6 months of age (Fig. 3). GD2 was intracellularly compartmentalized in caveolin-1-null NP cells but localized in the cell membrane of WT mice NCs. Moreover, the percentage of cells expressing nuclear pSmad2 protein was significantly higher in the 1.5-month-old murine caveolin-1-null NPs compared with murine WT NPs of the same age. The percentage of cells expressing pSmad2 was significantly increased in the 3-month-old WT NPs compared with the 1.5-month-old WT NPs. At 3 and 6 months of age, WT and caveolin-1-null NP cells showed a similar percentage of pSmad2-positive stained nuclei, indicating comparable levels of activated TGF-β/Smad2 signaling (*p* < 0.05) (Fig. 4).

### Caveolin-1 expression and apoptosis during canine IVD degeneration

**Fig. 5:**
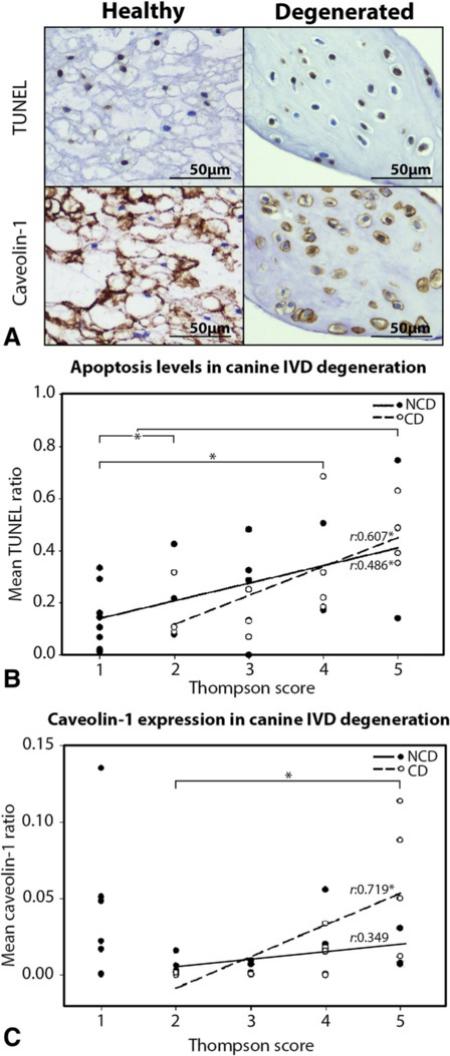
Caveolin-1 expression and apoptosis levels are increased during canine intervertebral disc (IVD) degeneration. **a** Terminal deoxynucleotidyl transferase deoxyuridine triphosphate nick-end (TUNEL) assay and immunohistochemical staining for caveolin-1. Part of a healthy canine nucleus pulposus (NP) (Thompson grade I) and a severely degenerated canine NP (Thompson grade V) with brown apoptotic notochordal cell (NC) nuclei (TUNEL) and brown NC membranes and cytoplasm (caveolin-1). **b** The mean TUNEL ratio (the proportion of positively stained TUNEL nuclei in the NP) per Thompson degeneration score of canine IVDs. Apoptosis levels increased during canine IVD degeneration in chondrodystrophic (CD) and nonchondrodystrophic (NCD) dogs. The *r* values indicate the partial correlation between the mean TUNEL ratio and IVD degeneration grade (Thompson grade range I–V) for CD and NCD dogs. *p < 0.05 (significance indicated for changes related to Thompson grade). **c** The mean caveolin-1 ratio (the proportion of the total NP surface area that stained positive for caveolin-1) per Thompson degeneration score of canine IVDs. The *r* values indicate the partial correlation between the mean caveolin-1 ratio and IVD degeneration grade (Thompson score range II–V) for CD and NCD dogs. Caveolin-1 protein expression increases during canine IVD degeneration in CD and NCD dogs. *p < 0.05 (significance indicated for changes related to Thompson grade). *n* = 37 IVD samples from 16 dogs

Apoptotic (TUNEL-positive) cell nuclei were observed in healthy NC-containing NPs and in degenerated CLC-containing NPs (Fig. 5a). NPs obtained from IVDs with Thompson grades IV and V demonstrated significantly more apoptotic cells than healthy and early degenerated NPs (Thompson grades I and II) in CD and NCD dogs (*p* < 0.05) (Fig. 5b). Also, there was a significant positive correlation between apoptosis levels and IVD degeneration grade in CD and NCD dogs (*r* = 0.607 and *r* = 0.486, respectively; *p* < 0.05) (Fig. 5b). There were no statistically significant differences between CD and NCD dogs for apoptosis levels per Thompson grade.

Caveolin-1 expression was localized in the cytoplasm and cell membranes of NCs from healthy NPs and in CLCs from (severely) degenerated NPs (Fig. 5a). No correlation between caveolin-1 expression and donor age was observed (data not shown). Also, there were no statistically significant differences between CD and NCD dogs for caveolin-1 expression per Thompson grade. The expression of caveolin-1 significantly increased during IVD degeneration in CD dogs (*p* < 0.05) (Fig. 5c). Caveolin-1 expression and IVD degeneration grade (Thompson grades I–V) did not correlate in NCD dogs (*r* = −0.247), while in CD dogs there was a strong positive correlation between caveolin-1 expression and IVD degeneration grade (*r* = 0.719, *p* < 0.01) (Fig. 5c). However, given that NC-rich Thompson grade I IVDs were available only from NCD dogs, when excluding those Thompson grade I IVDs, caveolin-1 expression weakly correlated with IVD degeneration for the range of Thompson grades II–V in the NCD dogs (*r* = 0.349) (Fig. 5c). No statistically significant correlation between caveolin-1 expression and apoptosis levels was found in CD or NCD canine NPs (data not shown).

### In vitro effects of caveolin-1 on human and canine CLCs

#### Effect of caveolin-1 silencing in canine CLCs in vitro

**Fig. 6:**
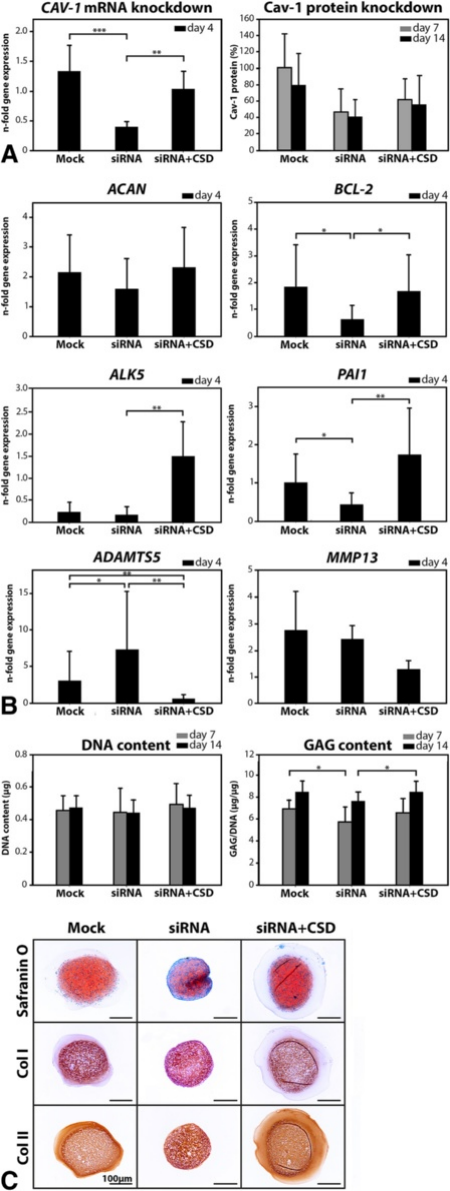
The effect of in vitro caveolin (Cav)-1 silencing on chondrocyte-like cells (CLCs) from chondrodystrophic dogs. In vitro Cav-1 silencing decreased transforming growth factor-β/Smad2/3 signaling and canine CLC extracellular matrix deposition, while 10 μM Cav-1 scaffolding domain peptide (CSD) supplementation rescued this effect. **a** Cav-1 messenger RNA (*n*-fold change ± SD) and protein knockdown by microaggregates silenced for Cav-1 (siRNA). The Cav-1 protein level in the mock-treated microaggregates at day 7 was set at 100 %. **b** Real-time quantitative polymerase chain reaction results (*n*-fold change ± SD). Gene expression in the nontreated CLC microaggregates was set at 1. **c** DNA and glycosaminoglycan (GAG) content (corrected for DNA content) of the canine CLC microaggregates with corresponding Safranin O/Fast Green, collagen type I, alpha 1 (Col I), and collagen type II, alpha 1 (Col II) immunohistochemically stained sections. Bars indicate 100 μm. *n* = 6. *ACAN* aggrecan, *BCL2* B-cell CLL/lymphoma 2, *ALK5* activin receptor-like kinase 5, *PAI1* plasminogen activator inhibitor 1, *ADAMTS5* a disintegrin and metalloproteinase with thrombospondin motifs 5, *MMP13* matrix metalloproteinase 13. *p < 0.05, **p < 0.01

With a clinical directive in mind, caveolin-1 was silenced and CSD peptide (which mimics the function of caveolin-1 [8, 9, 32–34]) was added to canine and human CLCs derived from degenerated IVDs to determine whether caveolin-1 modifies TGF-β signaling in CLCs with concurrent effects on ECM level. *CAV1* messenger RNA (mRNA) knockdown in the caveolin-1-silenced CLCs was 70 % compared with mock-treated CLCs at day 4 (*p* < 0.001) (Fig. 6a). CSD treatment upregulated *CAV1* mRNA expression in canine CLCs silenced for caveolin-1 (*p* < 0.05). Caveolin-1 protein knockdown was 53 % compared with mock-treated CLCs at day 7 and 48 % at day 14 (Fig. 6a). While *PAI1* expression was decreased in caveolin-1-silenced CLCs compared with mock-treated CLCs (*p* < 0.05), *ALK5* and *PAI1* expression were increased by CSD treatment of these CLCs (*p* < 0.01) (Fig. 6b), which indicated activated TGF-β/Smad2/3 signaling under the influence of CSD at least at the gene expression level. Expression of the antiapoptotic *BCL2* gene was decreased by caveolin-1 silencing, whereas the addition of CSD reversed this effect (*p* < 0.05) (Fig. 6b). Gene expression of *MMP13*, *ACAN* (Fig. 6b), *COL1A1*, *COL2A1*, *SOX9*, *TIMP1*, *ALK1*, *ID1*, *CCND1*, *BAX*, and *CASP3* (data not shown) was not significantly different between the conditions.

Caveolin-1-silenced CLCs showed increased *ADAMTS5* expression compared with mock-treated CLCs (*p* < 0.05), whereas CSD treatment reversed this effect (*p* < 0.01) (Fig. 6b). Also, the GAG content of caveolin-1-silenced microaggregates was significantly less than in mock-treated microaggregates at day 7 (17 %; *p* < 0.05), but not at day 14 (10 %) (Fig. 6c). CSD treatment increased the GAG content of caveolin-1-silenced microaggregates at day 14 (14 %; *p* < 0.05). GAG release into the culture medium (data not shown) and the DNA content (Fig. 6c) of the microaggregates was not considerably different between the conditions.

All CLC microaggregates stained positive for GAGs and collagen types I and II (Fig. 6c). In the mock-treated microaggregates, and mainly in the caveolin-1-silenced microaggregates supplemented with CSD, a collagen type II–positive rim was present, but not in the caveolin-1-silenced microaggregates not supplemented with CSD.

#### Dose–response effect of caveolin-1 peptide on canine and human CLCs in vitro

**Fig. 7:**
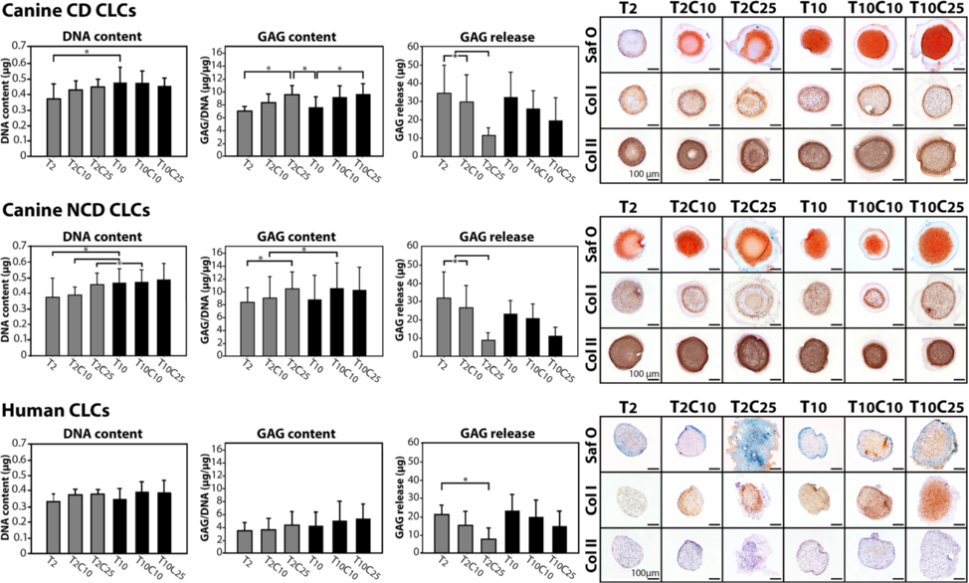
The dose–response effect of caveolin-1 on canine and human chondrocyte-like cells (CLCs) in vitro. DNA content, glycosaminoglycan (GAG) content (corrected for DNA content), and GAG release into the culture medium of human and chondrodystrophic (CD) and nonchondrodystrophic (NCD) canine CLC microaggregates with corresponding Safranin O (Saf O)/Fast Green collagen types I (Col I) and II (Col II) immunohistochemically stained sections. Generally, the GAG content was increased in the microaggregates treated with 25 μM caveolin-1 scaffolding domain (CSD), whereas the GAG release in the culture medium was decreased in these 25 μM CSD-treated microaggregates. *T*_*2*_ 2 ng/ml transforming growth factor (TGF)-β_1_, *T*_*10*_ 10 ng/ml TGF-β_1_, *C*_*10*_ 10 μM CSD, *C*_*25*_ 25 μM CSD. Bars indicate 100 μm. *n* = 5 (CD and NCD) and *n* = 3 (human). Two microaggregates were analyzed per donor and condition. *p < 0.05

Because CSD treatment rescued the effects of caveolin-1 silencing and exerted anabolic effects on canine CLCs by modifying TGF-β signaling at the gene expression level, different concentrations (i.e., 2 ng/ml TGF-β_1_ [T_2_], 10 ng/ml TGF-β_1_ [T_10_], 10 μM CSD [C_10_], and 25 μM CSD [C_25_]) were tested to determine the additive effect of CSD on TGF-β_1_ treatment of human, CD, and NCD canine CLCs. The DNA content of CD and NCD canine T_10_-treated microaggregates was significantly higher than that of T_2_-treated microaggregates (*p* < 0.05), whereas this was not observed in human microaggregates (Fig. 7). Only in NCD canine donors, T_2_C_25_-treated microaggregates showed an increased DNA content compared with T_2_C_10_-treated microaggregates (*p* < 0.05). CSD treatment did not affect the DNA content of human and CD canine microaggregates.

In CD and NCD donors, the GAG content of the T_2_C_25_-treated microaggregates was significantly higher (35 % for CD, 25 % for NCD) than the GAG content of T_2_-treated microaggregates (Fig. 7). Additionally, only in CD donors was the GAG content of the T_10_C_25_-treated microaggregates significantly higher (26 %) than the GAG content of the T_10_-treated microaggregates. GAG release in the culture medium was decreased by approximately three times in T_2_C_25_-treated CLC microaggregates compared with T_2_-treated CLC microaggregates in all species (*p* < 0.05) (Fig. 7). Total GAG production (GAG content in the microaggregates plus GAG release into the culture medium) appeared to be 20–40 % decreased by 25 μM CSD treatment in all species and was significantly decreased in T_2_C_25_-treated CD and NCD canine CLCs compared with T_2_- and T_2_C_10_-treated CD and NCD canine CLCs (*p* < 0.05), but not significantly different between the differentially treated human CLCs (data not shown). The GAG content of human CLC microaggregates was lower than the GAG content of CD and NCD canine microaggregates. In contrast to canine CLC microaggregates, human CLC microaggregates demonstrated no collagen type II protein deposition (Fig. 7). Furthermore, CSD treatment increased collagen type I deposition in human CLC microaggregates, whereas this was not observed in canine CLC microaggregates.

**Fig. 8:**
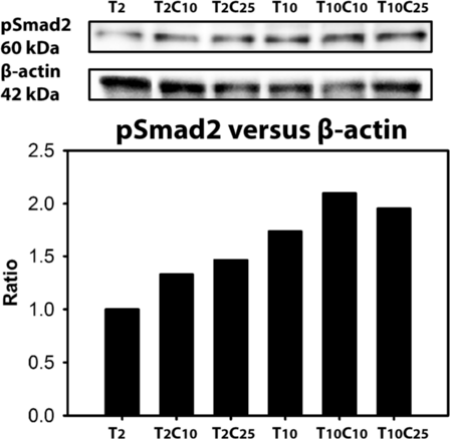
Caveolin-1 enhances transforming growth factor (TGF)-β/phosphorylated Smad2 (pSmad2) signaling in canine chondrocyte-like cells (CLCs) in vitro. Western blot analysis for pSmad2 and β-actin protein expression in chondrodystrophic (CD) canine CLCs after 24 h of treatment, expressed as pSmad2/β-actin ratio. *T*_*2*_ 2 ng/ml TGF-β_1_, *T*_*10*_ 10 ng/ml TGF-β_1_, *C*_*10*_ 10 μM CSD, *C*_*25*_ 25 μM CSD. The pSmad2/β-actin ratio in the T_2_-treated CLCs was set at 1

Western blot analysis demonstrated that pSmad2 protein expression was 1.7 times higher in CD canine CLCs treated with 2 ng/ml TGF-β_1_ than in those treated with 10 ng/ml TGF-β_1_ (Fig. 8). pSmad2 protein expression was 1.3–1.5 times higher in the canine CLCs treated with 10 or 25 μM CSD and 2 ng/ml TGF-β_1_ than in those treated with only 2 ng/ml TGF-β_1_ (Fig. 8). Furthermore, pSmad2 protein expression was 1.1–1.2 times higher in the CLCs treated with 10 or 25 μM CSD and 10 ng/ml TGF-β_1_ than in those treated with only 10 ng/ml TGF-β_1_ (Fig. 8). Altogether, this indicates that CSD peptide treatment enhanced TGF-β/pSmad2 signaling.

## Discussion

The present study demonstrates that caveolin-1 plays a role in IVD degeneration. In vivo studies with murine caveolin-1-null NPs demonstrated that caveolin-1 was essential for NC preservation. Furthermore, we have demonstrated that caveolin-1 expression and apoptosis levels were significantly increased during CD canine IVD degeneration. In addition, in vitro caveolin-1 silencing decreased CLC ECM deposition, while CSD supplementation increased TGF-β/pSmad2 signaling at the gene and protein expression levels and enhanced the anabolic effects of TGF-β_1_ on CLCs, resulting in higher ECM deposition. Taken together, this may indicate that the increased caveolin-1 expression during IVD degeneration facilitates an anabolic reparative response of the CLCs at least by modifying TGF-β signaling rather than being merely correlated with CLC apoptosis.

### Caveolin-1 is essential for NC physiology

Histological analysis of caveolin-1-null NPs revealed that apoptotic cells were present in the healthy WT murine NP at 1.5, 3, and 6 months of age, implying that during IVD tissue homeostasis, there is physiologic turnover of NP cells (they proliferate and undergo apoptosis). Affirmatively, previous work already demonstrated apoptotic cells in IVDs from 2-week-old WT mice [35]. Caveolin-1-null NPs showed the absence of typical NCs in favor of chondrocyte/fibroblast-like cells with an increased apoptotic activity, suggesting that caveolin-1 is essential for NC preservation. In line with this thought, NPs of NCD dogs with Thompson grade I, rich in NC cells, abundantly expressed caveolin-1, while the caveolin-1-null mice had lost their NCs already when they were skeletally mature (young adult), which is rather comparable with the loss of NCs in young adult CD dogs and humans. In the NP of 1.5-month-old caveolin-1-null mice (with lower total NP cells numbers than WT mice), Tie2^+^/GD2^+^ progenitor cells were present, and TGF-β signaling mediated by pSmad2 probably facilitated abundant ECM deposition. Affirmatively, Tie2^+^/GD2^+^ NP cells have been shown to possess superior ECM production, self-renewal, multipotent differentiation, and proliferation capacity [31]. Increased cell proliferation is associated with late-stage IVD degeneration [36]. Notably, the NPPC proliferation marker GD2 was expressed in caveolin-1-null and WT mice NPs, but Ki-67 positivity was not detected. The short Ki-67 protein half-life (90 minutes) and nutritionally deprived NP environment may explain the absence of Ki-67 in murine NPs [36]. GD2 appeared intracellularly compartmentalized in caveolin-1-null NP cells but localized to the cell membrane of WT NCs. We hypothesize that in the caveolin-1-null NP phenotype, GD2 may not mark proliferating cells per se if the different localization could render GD2 inactive, but researchers in future studies should look into the functionality of GD2 in caveolin-1-null NP cells.

Altogether, caveolin-1 plays a role in the preservation of healthy NCs because in vivo inactivation of caveolin-1 induced a change in cell phenotype from NCs toward CLCs with high apoptotic activity in mice. Activated NPPCs and TGF-β/pSmad2 signaling probably resulted into GAG- and collagen-rich ECM deposition in the caveolin-1-null mice NP. The caveolin-1-null mice showed several metabolic/endocrine disorders [37–39] that could have influenced their NP phenotype (e.g., insulin resistance leading to diabetes mellitus, which has been associated with premature NC senescence and apoptosis) [40]. Further studies need to be concentrated at earlier stages of development to delineate how caveolin-1 preserves NCs, including modification of mitogen-activated protein kinase/extracellular signal-regulated kinase, Wnt [15], Indian hedgehog and/or sonic hedgehog signaling pathways [41, 42], and/or glucose transport [43, 44].

### Caveolin-1 increases during IVD degeneration, mediating a reparative response

Caveolin-1 is known to induce apoptosis via—among others—cell cycle arrest in the G_2_/M phase [45] and by influencing the expression of the antiapoptosis protein survivin [46, 47]. Caveolin-1 may induce senescence in CLCs, given that, in NPs derived from degenerated CD canine and human IVDs, caveolin-1 expression [16] and apoptosis levels were positively correlated with IVD degeneration grade. However, a causative relationship in the IVD remains to be determined. On the basis of the results of the present study, it is tempting to hypothesize that the increased caveolin-1 expression in degenerated IVDs may be part of an ultimate attempt at (unsuccessful) repair. With a clinical directive in mind (degenerated human and canine IVDs contain almost 100 % CLCs), the effect of caveolin-1 was investigated in human and canine CLCs derived from degenerated IVDs by silencing for caveolin-1 and/or supplementing with CSD peptide. Caveolin-1 silencing decreased antiapoptotic *BCL2* expression, whereas CSD addition increased it to baseline levels again, opposing the hypothesis that caveolin-1 itself induces CLC apoptosis. Furthermore, caveolin-1 silencing increased *ADAMTS*5 expression and decreased the GAG content of the microaggregates, whereas when the canine CLCs were treated with CSD, *ADAMTS5* expression decreased and the GAG content was restored. In line with this, GAG release was decreased in 25 μM CSD-treated human and canine CLCs. Notably, caveolin-1 silencing and CSD supplementation did not influence collagen type I deposition by canine CLCs, while its deposition was increased by CSD treatment in human CLCs. It is plausible that caveolin-1 exerts a protective effect by decreasing ECM degradation, given its well-described anti-inflammatory properties [48–50]. This altogether implies that decreased GAG degradation resulted in the significantly increased GAG content of CSD-treated canine CLCs. The underlying mechanisms for the latter could be decreased ECM degradation and/or more collagen type II protein expression that enables the deposition [51] and prevents the release of GAGs.

The results of this study imply that caveolin-1 regulates TGF-β signaling in CLCs in vitro, because caveolin-1 silencing decreased, whereas CSD supplementation increased TGF-β/Smad2 signaling at the gene and protein expression levels. TGF-β signals through two membrane TGF-β receptors type I (ALK1 and ALK5) and one TGF-β receptor type II. These TGF-β receptors have been demonstrated to colocalize with caveolin-1 [20]. TGF-β receptor type II phosphorylates TGF-β receptor type I. When ALK5 is phosphorylated, Smad2/3 signaling becomes activated and ECM is produced. However, when ALK1 is phosphorylated, Smad1/5/8 signaling becomes activated, which inhibits Smad2/3 signaling and ECM production [52, 53]. In this study, we have demonstrated that CSD enhanced the anabolic effect of TGF-β_1_ in terms of ECM deposition in the microaggregates. Caveolin-1 silencing decreased *PAI1* (a classical readout for activated Smad2/3 TGF-β signaling [27–29]) gene expression, while CSD supplementation increased *ALK5* and *PAI1* gene expression, did not influence *ALK1* or *ID1* (readout for activated Smad1/5/8 signaling) gene expression, and increased pSmad2 protein expression. This all indicates that caveolin-1 indeed enhanced TGF-β/Smad2/3 signaling.

There is evidence that caveolin-1 acts differently according to stimulating and/or inhibiting signals and cellular context (e.g., cell type, age, and disease and/or health stage of the tissue) [7, 11, 54]. In the present study, caveolin-1 enhanced TGF-β signaling mediated by pSmad2 in CLCs from degenerated IVDs in vitro, while the opposite was true in earlier reports [14, 55–57]. In line with our results, however, TGF-β signaling is reduced in caveolin-1-deficient fibroblasts [58] and regenerating hepatocytes of caveolin-1-null mice [20]. Notably, in caveolin-1-silenced canine CLCs, ECM production was decreased, whereas in caveolin-1-null mice, which initially contained primarily NCs, abundant ECM was deposited. This implies that caveolin-1 may indeed have different functions in NCs and CLCs, because caveolin-1 is required for NC preservation, whereas in CLCs it may be related to apoptosis and/or it may influence signaling pathways (such as TGF-β) that support ECM deposition.

## Conclusions

The present study demonstrates that caveolin-1 plays a role in IVD degeneration. The abundant expression of caveolin-1 in NCs from NCD dogs and the loss of healthy NCs in the NP of caveolin-1-null mice indicate that caveolin-1 supports preservation of the NC phenotype. In the CD canine NP, both caveolin-1 expression and apoptosis levels had a positive correlation with IVD degeneration grade, indicating that caveolin-1 may be related with CLC apoptosis. Nevertheless, ECM deposition was decreased by in vitro caveolin-1 silencing of canine CLCs, whereas CSD peptide increased TGF-β/pSmad2 signaling at the gene and protein expression levels and enhanced the anabolic effects of TGF-β_1_ with concomitant increased ECM deposition. This implies that the increased caveolin-1 expression during IVD degeneration may be an anabolic repair response rather than merely being correlated with CLC apoptosis. Further studies are needed to investigate how caveolin-1 modifies other signaling pathways and facilitates IVD repair.

## Additional files

Additional file 1: Cytospins of canine CLC donors. (DOCX 1497 kb) Additional file 2: Overview of the primer pairs. (DOCX 25 kb) Additional file 3: Morphometry of murine IVDs. (DOCX 499 kb)

## Abbreviations

Ab: antibody
ACAN: aggrecan
ADAMTS5: a disintegrin and metalloproteinase with thrombospondin motifs 5
ALK1: activin receptor-like kinase 1
ALK5: activin receptor-like kinase 5
BAX: B-cell CLL/lymphoma 2–associated protein
BCL2: B-cell CLL/lymphoma 2
BSA: bovine serum albumin
*CASP3*: caspase 3
*CAV1*: caveolin-1
*CCND1*: cyclin D1
CD: chondrodystrophic
CLC: chondrocyte-like cell
*COL1A1*: collagen type I, alpha 1
*COL2A1*: collagen type II, alpha 1
CSD: caveolin-1 scaffolding domain
ECM: extracellular matrix
GAG: glycosaminoglycan
*GAPDH*: glyceraldehyde 3-phosphate dehydrogenase
GC: guanine-cytosine
GD2: disialoganglioside 2
*HPRT*: hypoxanthine-guanine phosphoribosyltransferase
*ID1*: inhibitor of DNA binding 1
IgG: immunoglobulin G
IVD: intervertebral disc
MMP13: matrix metalloproteinase 13
mRNA: messenger RNA
NC: notochordal cell
NCD: nonchondrodystrophic
NP: nucleus pulposus
NPPC: nucleus pulposus progenitor cell
*PAI1*: plasminogen activator inhibitor 1
PBS: phosphate-buffered saline
pSmad2: phosphorylated Smad2
RIPA: radioimmunoprecipitation assay
*RPS19*: ribosomal protein S19
*SDHA*: succinate dehydrogenase subunit A
siRNA: silencing RNA
*SOX9*: (sex determining region Y)-box 9
TGF: transforming growth factor
*TIMP1*: tissue inhibitor of metalloproteinase 1
TUNEL: terminal deoxynucleotidyl transferase deoxyuridine triphosphate nick-end labeling
WT: wild type
