# Supplementary material for: Increased caveolin-1 in intervertebral disc degeneration facilitates repair
Source: Arthritis Res Ther. 2016 Mar 3;18:59. doi: 10.1186/s13075-016-0960-y (PMC4778307; doi:10.1186/s13075-016-0960-y)
Supplement: Additional file 1: — Cytospins of canine CLC donors. (DOCX 1497 kb) [file 13075_2016_960_MOESM1_ESM.docx]

**Additional file 1. Cytospins of canine CLC donors**


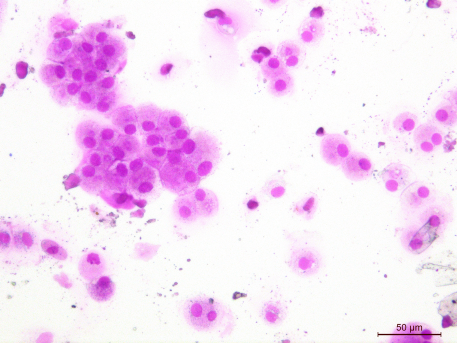

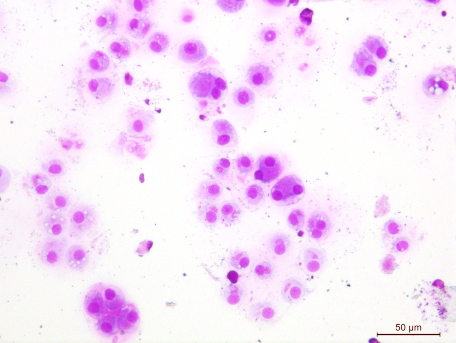
 **
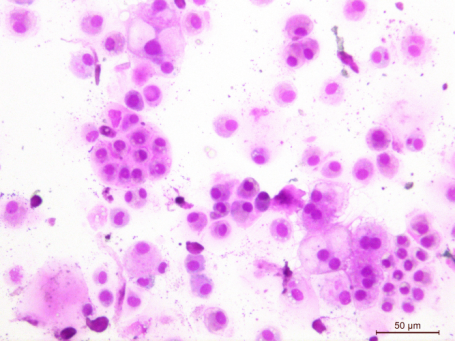
**

**
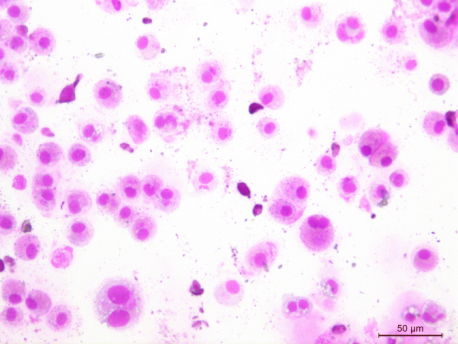

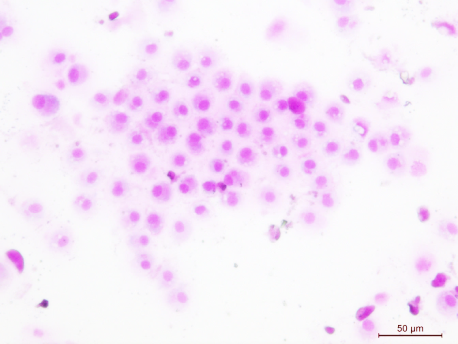
**

Cytospins of the 5 canine NCD donors used in the study: 100% CLCs were present, and no NCs with the typical vacuolated appearance.
