# Supplementary material for: Increased caveolin-1 in intervertebral disc degeneration facilitates repair
Source: Arthritis Res Ther. 2016 Mar 3;18:59. doi: 10.1186/s13075-016-0960-y (PMC4778307; doi:10.1186/s13075-016-0960-y)
Supplement: Additional file 2: — Overview of the primer pairs. (DOCX 25 kb) [file 13075_2016_960_MOESM2_ESM.docx]

**Additional file 2. Primers used for quantitative PCR of canine samples**

| **Genes** | **Forward sequence 5’ → 3’** | **Reverse sequence 5’ → 3’** | **Amplicon size** | **Annealing temp (°C)** | **Accession no.** |
| --- | --- | --- | --- | --- | --- |
| **Reference genes** |  |  |  |  |  |
| *GAPDH* | TGTCCCCACCCCCAATGTATC | CTCCGATGCCTGCTTCACTACCTT | 100 | 58 | NM_001003142 |
| *HPRT* | AGCTTGCTGGTGAAAAGGAC | TTATAGTCAAGGGCATATCC | 104 | 58 | NM_001003357 |
| *RPS19* | CCTTCCTCAAAAAGTCTGGG | GTTCTCATCGTAGGGAGCAAG | 95 | 61 | XM_005616513 |
| *SDHA* | GCCTTGGATCTCTTGATGGA | TTCTTGGCTCTTATGCGATG | 92 | 56.5 | XM_535807 |
| **Target genes** |  |  |  |  |  |
| *ACAN* | GGACACTCCTTGCAATTTGAG | GTCATTCCACTCTCCCTTCTC | 111 | 62 | XM_005618252 |
| *ADAMTS5* | CTACTGCACAGGGAAGAG | GAACCCATTCCACAAATGTC | 149 | 61 | XM_846025.3 |
| *ALK1* | CCTTTGGTCTGGTGCTGTG | CGAAGCTGGGATCATTGGG | 107 | 61 | XM_534796.4 |
| *ALK5* | GAGGCAGAGATTTATCAGACC | ATGATAATCTGACACCAACCAG | 116 | 59.5 | NC_006593.3 |
| *BAX* | CCTTTTGCTTCAGGGTTTCA | CTCAGCTTCTTGGTGGATGC | 108 | 58 | NM_001003011.1 |
| *BCL2* | TGGAGAGVGTCAACCGGGAGATGT | AGGTGTGCAGATGCCGGTTCAGGT | 87 | 62 | NM_001002949 |
| *CASP3* | ATCACTGAAGATGGATGGGTTGGGTT | TGAAAGGAGCATGTTCTGAAGTAGCACT | 139 | 58 | NM_001003042 |
| *CAV1* | CGCACACCAAGGAAATCG | AAATCAATCTTGACCACGTCG | 72 | 60 | NM_001003296 |
| *COL1A1* | GTGTGTACAGAACGGCCTCA | TCGCAAATCACGTCATCG | 109 | 61 | NM_001003090 |
| *COL2A1* | GCAGCAAGAGCAAGGAC | TTCTGAGAGCCCTCGGT | 151 | 62 | XM_005636674 |
| *CCND1* | GCCTCGAAGATGAAGGAGAC | CAGTTTGTTCACCAGGAGCA | 117 | 60 | NM_001005757.1 |
| *ID1* | CTCAACGGCGAGATCAG | GAGCACGGGTTCTTCTC | 135 | 59.5 | XM_847117.2 |
| *MMP13* | CTGAGGAAGACTTCCAGCTT | TTGGACCACTTGAGAGTTCG | 250 | 65 | XM_536598 |
| *PAI1* | AAACCTGGCGGACTTCTC | ACTGTGCCACTCTCATTCAC | 98 | 61.5 | NM_001197095 |
| *SOX9* | CGCTCGCAGTACGACTACAC | GGGGTTCATGTAGGTGAAGG | 105 | 62 | NM_001002978 |
| *TIMP1* | GGCGTTATGAGATCAAGATGAC | ACCTGTGCAAGTATCCGC | 120 | 66 | NM_001003182 |

All primers were designed in-house using Perlprimer except for MMP-13 [1] and Bax [2].

**References**

1. Muir P, Danova NA, Argyle DJ, Manley PA, Hao Z: **Collagenolytic protease expression in cranial cruciate ligament and stifle synovial fluid in dogs with cranial cruciate ligament rupture.** *Vet Surg* 2005, **34**(5)**:**482-490.

2. Mahmoudabady M, Niazmand S, Shafei MN, McEntee K: **Investigation of apoptosis in a canine model of chronic heart failure induced by tachycardia.** *Acta Physiol Hung* 2013, **100**(4)**:**435-444.
