# Supplementary material for: Increased caveolin-1 in intervertebral disc degeneration facilitates repair
Source: Arthritis Res Ther. 2016 Mar 3;18:59. doi: 10.1186/s13075-016-0960-y (PMC4778307; doi:10.1186/s13075-016-0960-y)
Supplement: Additional file 3: — Morphometry of murine IVDs. (DOCX 499 kb) [file 13075_2016_960_MOESM3_ESM.docx]

**Additional file 3. Histomorphometrical measurements of the IVD, AF, and NP of WT and caveolin-1 null mice**


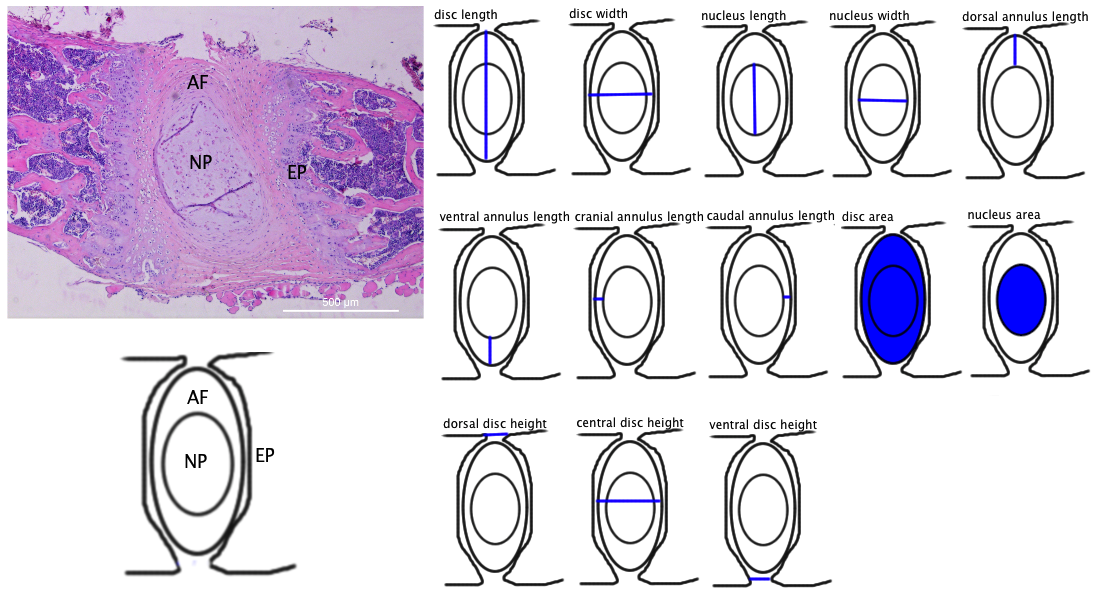


| Measurement | WT 1.5 months | Cav-1 null 1.5 months | WT 3 months | Cav-1 null 3 months | WT 6 months | Cav-1 null 6 months |
| --- | --- | --- | --- | --- | --- | --- |
| IVD length (µm) | 915 ± 207 | 964 ± 152 | 1,139 ± 151 | 1,149 ± 55 | 1,209 ± 251 | 984 ± 111 |
| IVD width (µm) | 359 ± 51 | 383 ± 53 | 356 ± 42 | 376 ± 80 | 347 ± 50 | 391 ± 45 |
| Dorsal IVD height (µm) | 298 ± 57 | 349 ± 113 | 313 ± 50 | 235 ± 71 | 306 ± 40 | 297 ± 23 |
| Ventral IVD height (µm) | 508 ± 124 | 463 ± 148 | 570 ± 150 | 554 ± 80 | 482 ± 75 | 495 ± 89 |
| Central IVD height (µm) | 359 ± 51 | 383 ± 55 | 356 ± 41 | 377 ± 78 | 364 ± 51 | 391 ± 41 |
| IVD area (µm^2^) | 343,231 ± 89,733 | 398,678 ± 95,096 | 431,116 ± 105,283 | 402,970 ± 74,510 | 440,733 ± 130,238 | 358,400 ± 77,423 |
| NP length (µm) | 597 ± 196 | 520 ± 183 | 738 ± 75 | 671 ± 123 | 817 ± 213 | 584 ± 78 |
| NP width (µm) | 248 ± 77 | 294 ± 119 | 337 ± 39 | 360 ± 108 | 346 ± 50 | 349 ± 87 |
| NP area (µm^2^) | 134,884 ± 84,690 | 138,704 ± 77,067 | 200,078 ± 37,215 | 193,994 ± 75,365 | 234,358 ± 82,046 | 168,230 ± 49,878 |
| Dorsal AF length (µm) | 134 ± 56 | 264 ± 133 | 119 ± 32 | 177 ± 107 | 127 ± 64 | 190 ± 55 |
| Ventral AF length (µm) | 178 ± 62 | 179 ± 39 | 278 ± 64 | 293 ± 57 | 258 ± 52 | 201 ± 52 |
| Cranial AF length (µm) | 38 ± 31 | 49 ± 52 | 6.5 ± 5.7 | 8.1 ± 14.0 | 0 ± 0 | 18 ± 24 |
| Caudal AF length (µm) | 69 ± 53 | 38 ± 29 | 12.4 ± 18.4 | 6.7 ± 11.7 | 0 ± 0 | 23 ± 33 |

*Histomorphometrical measurements of the murine intervertebral disc (IVD). No statistically significant histomorphometrical differences in the IVD/NP/AF between WT and caveolin-1 null mice at 1.5, 3, and 6 months of age. WT: wild type, IVD: intervertebral disc, NP: nucleus pulposus, AF: annulus fibrosus*. IVD morphometry was performed on the raw images using Image J software (Rasband NIH).
